# Complexities in Hoffa Fracture Management: A Case Report on Diagnosis and Fixation Technique

**DOI:** 10.1155/cro/6459565

**Published:** 2026-03-08

**Authors:** Rohan Ratra, Anand. K Goyal

**Affiliations:** ^1^ Department of Trauma and Orthopaedics Subharti Medical College, Gautam Buddha Chikitsa Mahavidyalaya, Dehradun, India

## Abstract

**Introduction:**

Hoffa fractures, accounting for 0.65% of femoral fractures, are rare injuries to the distal femur, typically affecting the lateral condyle. These fractures are often overlooked in radiographs and pose challenges in classification, as traditional systems like Letenneur′s may not adequately address certain unique fracture patterns, as seen in the present case.

**Case Presentation:**

A 25‐year‐old male with trauma to the left knee and pelvis was diagnosed with a pelvic open‐book injury and a coronal split Hoffa fracture of the lateral femoral condyle. Surgical fixation involved external fixation for the pelvis and a posterolateral approach for the knee fracture, followed by a nonweight‐bearing regimen and rehabilitation exercises.

**Results:**

The patient showed significant improvement in both range of motion (ROM) and functional outcomes over follow‐up visits. Flexion increased from 25° preoperatively to 125° at 6 months, and the Neer score improved from 30 to 90, indicating an excellent outcome (*p* value–0.0077 for Neer score, 0.0144 for ROM).

**Discussion:**

Hoffa fractures are often missed on standard radiographs and require CT for accurate diagnosis. Existing classification systems offer limited guidance, as these fractures can present with varied patterns. In our case, a unique fracture pattern necessitated a posterolateral approach with lag screws from posterior to anterior, ensuring a proper reduction and fixation, in line with the principles of anatomical reduction and early mobilization.

**Conclusion:**

Hoffa fractures are challenging to diagnose and treat due to their atypical patterns. Preoperative CT planning and flexible surgical approaches, such as our use of posterior‐to‐anterior lag screws, are crucial. Focusing on anatomical reduction and early mobilization can optimize recovery, with further research needed to refine classifications and improve outcomes.

## 1. Introduction

Hoffa′s fracture refers to a specific type of fracture at the distal end of the femur, occurring within the joint and oriented in the coronal plane. It represents approximately 0.65% of all femoral fractures [1]. Friedrich Busch was the first to describe the condition in 1869 [2]. Later, in 1904, Albert Hoffa provided a more comprehensive and detailed account of the condition, expanding on Busch′s initial observations [3]. Hoffa fracture is classified as Type 33B3 fracture as per AO/OTA Muller classification [4]. Hoffa fractures and Type 33B fractures can impact either of the femoral condyles; however, there is a stronger tendency for them to occur in the lateral condyle. This is primarily due to the natural valgus alignment of the knee joint and the direction of the force applied during the injury. Interestingly, Hoffa fractures are frequently overlooked on standard radiographs, with studies indicating that around 30% of such fractures go undiagnosed in initial imaging. Furthermore, in cases involving Type 33B fractures, the occurrence of Hoffa fractures is observed in approximately 38% of patients [5].

In 1978, Letenneur categorized these fractures into three types based on the distance of the fracture line from the posterior femoral cortex and its orientation [6]. Although the Letenneur classification provides insight into the mechanism of injury, it does not offer guidance on the management of these fractures. Although it is a straightforward system, numerous fracture types have been identified that cannot be classified within the Letenneur framework. Various CT classification systems for Hoffa fractures have been outlined in the existing literature. In 2019, Bagaria et al. proposed a classification that divides these fractures into four distinct types based on the fracture pattern, with the fourth type being further subdivided into four subtypes to account for more specific fracture characteristics [7]. A year later, in 2020, Chandrabose et al. [8] published their own classification system, which categorizes Hoffa fractures into four types based on the nature of the fracture pattern, specifically whether it is simple, involves articular comminution, or includes posterior comminution.

In the present case, we are reporting a Hoffa fracture that does not align with the traditional Letenneur classification. Although it may potentially be classified as Type 1 according to Bagaria′s system or Type A in the classification proposed by Chandrabose et al., the fracture in question presents unique challenges. It is located extremely low, affecting nearly the entire posterior femoral condyle′s articular surface. This extensive involvement of the posterior condyle creates significant difficulty in both fixation and treatment, which is why we have chosen to report this particular case. The complexity of the fracture pattern and its impact on treatment options underscore the need for a detailed analysis and tailored management approach.

## 2. Case Presentation

**Figure 1:**
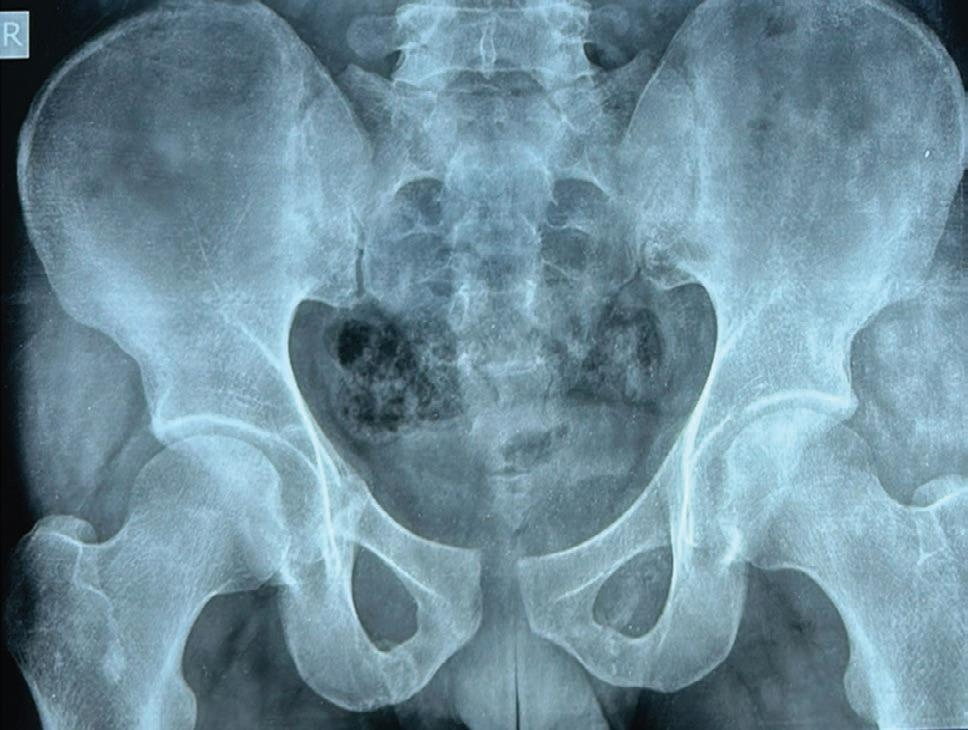
Anteroposterior (AP) pelvic radiograph showing a classic open‐book pelvic injury. There is pubic diastasis and fractures of the superior and inferior pubic rami on the right side. Images are oriented with anterior structures at the top.

A 25‐year‐old male patient presented to our accident and emergency (A&E) department after sustaining injuries in an accident involving trauma to his left knee and pelvis. On initial assessment, the patient was in significant pain and displayed difficulty with movement, particularly in the lower extremities. His vital signs were stable, but he showed signs of distress due to the nature of his injuries. On physical examination, there was notable tenderness and swelling around the left knee, with limited range of motion (ROM) due to pain. There was also bruising and a slight deformity around the pelvic region, with palpable tenderness over the pubic symphysis and right pelvic rim. The patient had difficulty bearing weight on his left leg and exhibited a positive log roll test, indicating a potential pelvic instability. Pelvic x‐rays revealed a classic open‐book pelvic injury, marked by pubic diastasis and fractures of the superior and inferior pubic rami on the right side (Figure 1). The knee radiograph demonstrated a coronal split fracture of the lateral femoral condyle on the lateral view; however, preoperative knee radiographs could not be retrieved for inclusion in this case report. Given the complexity of the injuries, further imaging was arranged to allow accurate characterization and appropriate management of both the pelvic and knee fractures. Computed tomography of the knee, including multiplanar reconstructions, revealed a solitary tangential fracture line without evidence of articular comminution involving the lateral femoral condyle. The fracture fragment was notably large, with the fracture line extending distally through the posterior aspect of the lateral femoral condyle (Figure 2). Although a preoperative knee radiograph was unavailable, computed tomography with multiplanar reconstructions provided a sufficient diagnostic detail. Nevertheless, the absence of standard radiographic views remains a limitation of this report.

Figure 2Preoperative computed tomography (CT) images of the right knee demonstrating a Hoffa fracture involving the lateral femoral condyle. (a) Coronal plane and (b) sagittal plane. Measurements were obtained using CT digital calipers; scale corresponds to 10 mm.

**Figure:**
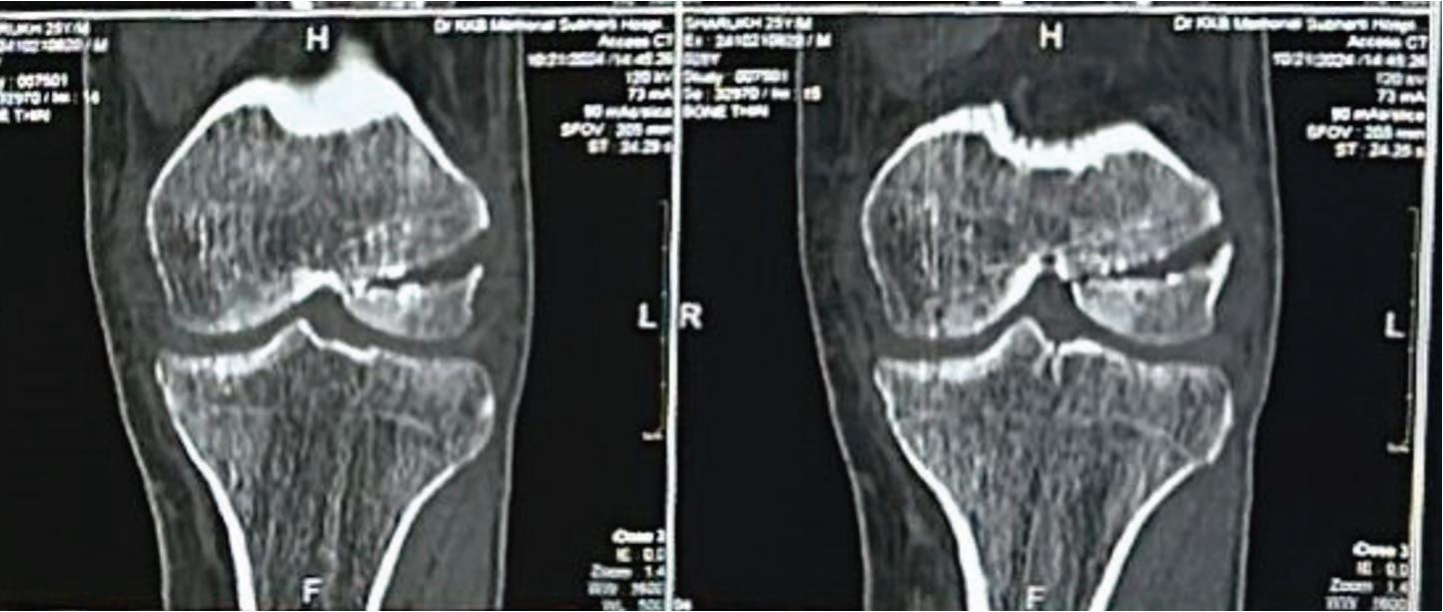
(a)

**Figure:**
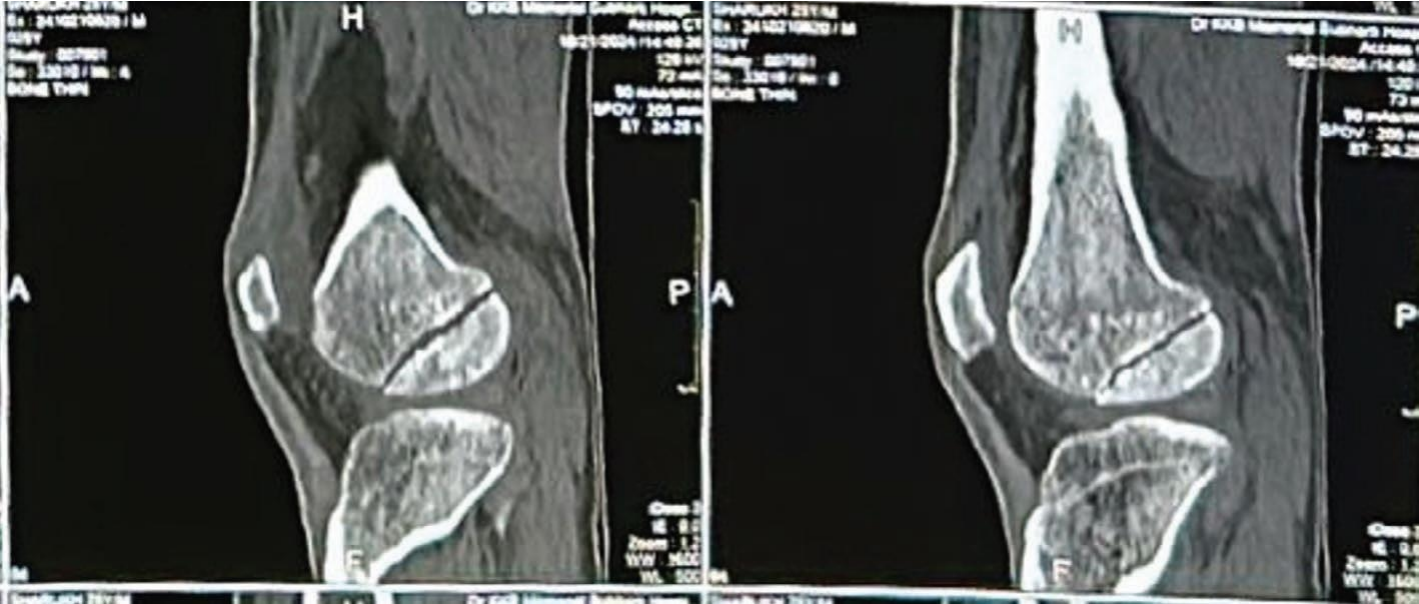
(b)

Measurements done on CT imaging demonstrated a coronal plane fracture involving approximately 41% of the posterior lateral femoral condyle. The fracture fragment measured 2.94 × 3.84 cm, whereas the overall condylar dimensions were 5 × 5.47 cm. The fracture line passed coronally near the junction of the posterior femoral condyle and femoral shaft, with a fragment size exceeding 2.5 cm from the posterior‐most point of the condyle, consistent with a Type 1 Hoffa fracture as per the classification proposed by Bagaria et al., [7].

**Figure 3:**
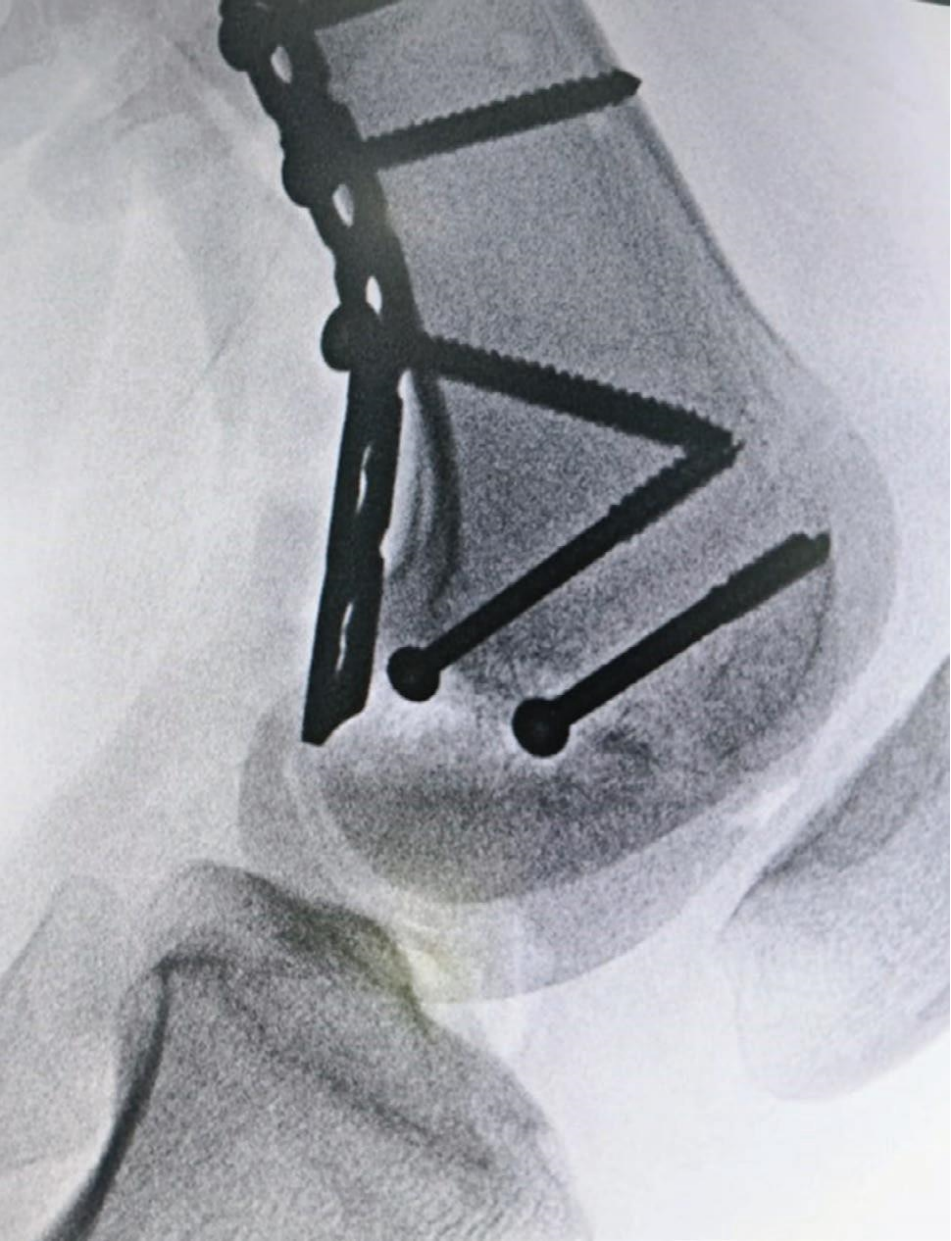
Intraoperative anteroposterior (AP) and lateral radiographs of the right distal femur demonstrating persistent fracture displacement after insertion of two cannulated screws. Image is oriented with anterior structures to the right.

After initial stabilization, the patient was scheduled for surgical fixation of both fractures the following day. The pelvic injury was managed using an external fixator, secured with two periacetabular pins. For the Hoffa′s fracture, a posterolateral approach was employed to access the fracture site. A longitudinal incision was made between the iliotibial band (IT band) and the biceps femoris, extending distally toward the joint line and toward the anterior aspect of the fibular head. The common peroneal nerve was carefully identified and retracted. Dissection was carried out through the interval between the biceps femoris posteriorly and the IT band anteriorly. The joint capsule was then elevated subperiosteally, from posterior to anterior. A tear in the capsule was already present, allowing a clear identification of the fracture. The fracture fixation was performed according to the initial 3D CT plan, which involved the use of two lag screws placed from posterior to anterior, given the fracture′s predominantly posterior orientation. However, after inserting the two screws, the fracture still appeared displaced (Figure 3). Due to the fracture′s distal location and its involvement with the articular surface of the posterior femoral condyle, a third screw was inserted as distally as possible. This third screw resulted in a satisfactory reduction in both the AP and lateral views (Figure 4). In this case, appropriate‐sized headless compression screws were not available at the time of surgery. Therefore, standard cannulated cancellous screws were used. Given the close proximity of the fracture line to the joint surface, meticulous countersinking was performed during screw insertion to avoid hardware prominence and minimize the risk of articular surface irritation. Care was taken to ensure that the screw heads were flush with or slightly recessed into the posterior cortical bone. Additionally, a contoured plate was applied over the posterior aspect of the lateral femoral condyle, proximal to the fracture line. Although no screws were inserted through the distal end of the plate due to its close proximity to the joint surface, the plate served as a posterior buttress.

Figure 4Intraoperative radiographs of the right distal femur showing insertion of the third cannulated screw. (a) Anteroposterior (AP) view, oriented with anterior structures at the top and medial structures to the left; (b) lateral view, oriented with anterior structures to the right and distal femur at the bottom.

**Figure:**
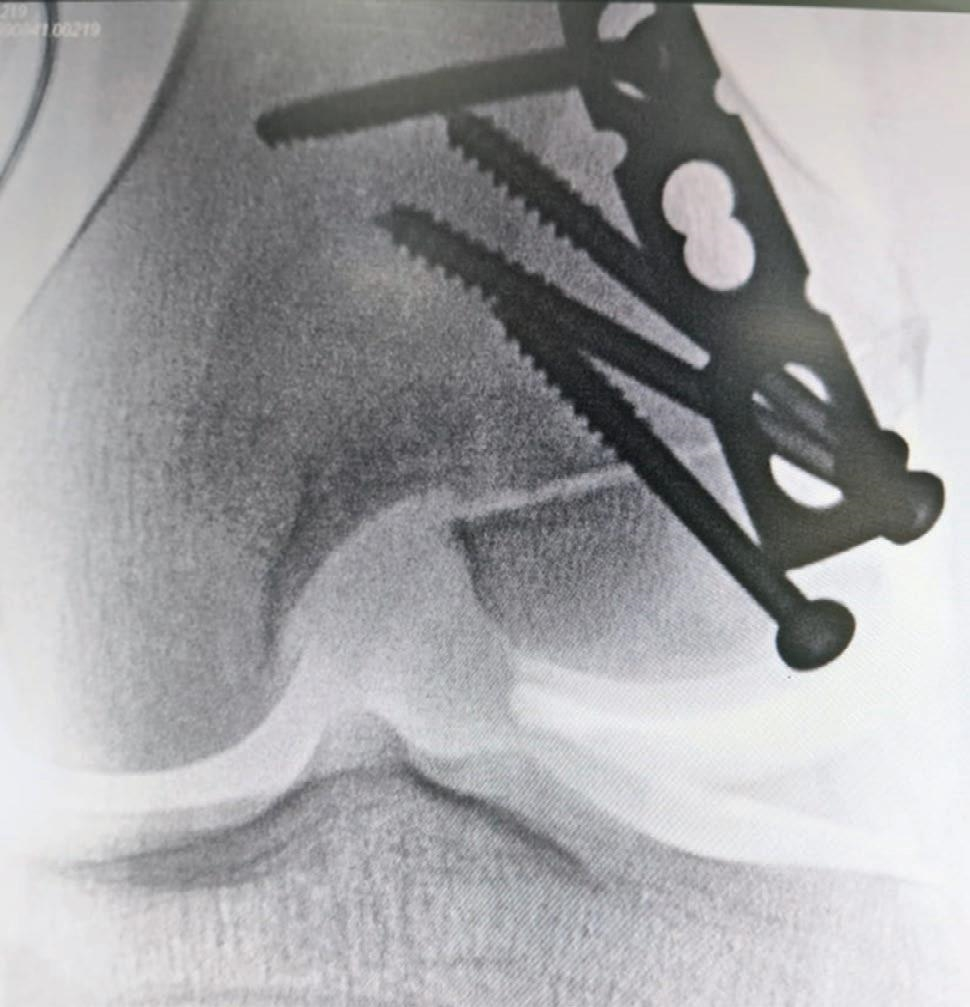
(a)

**Figure:**
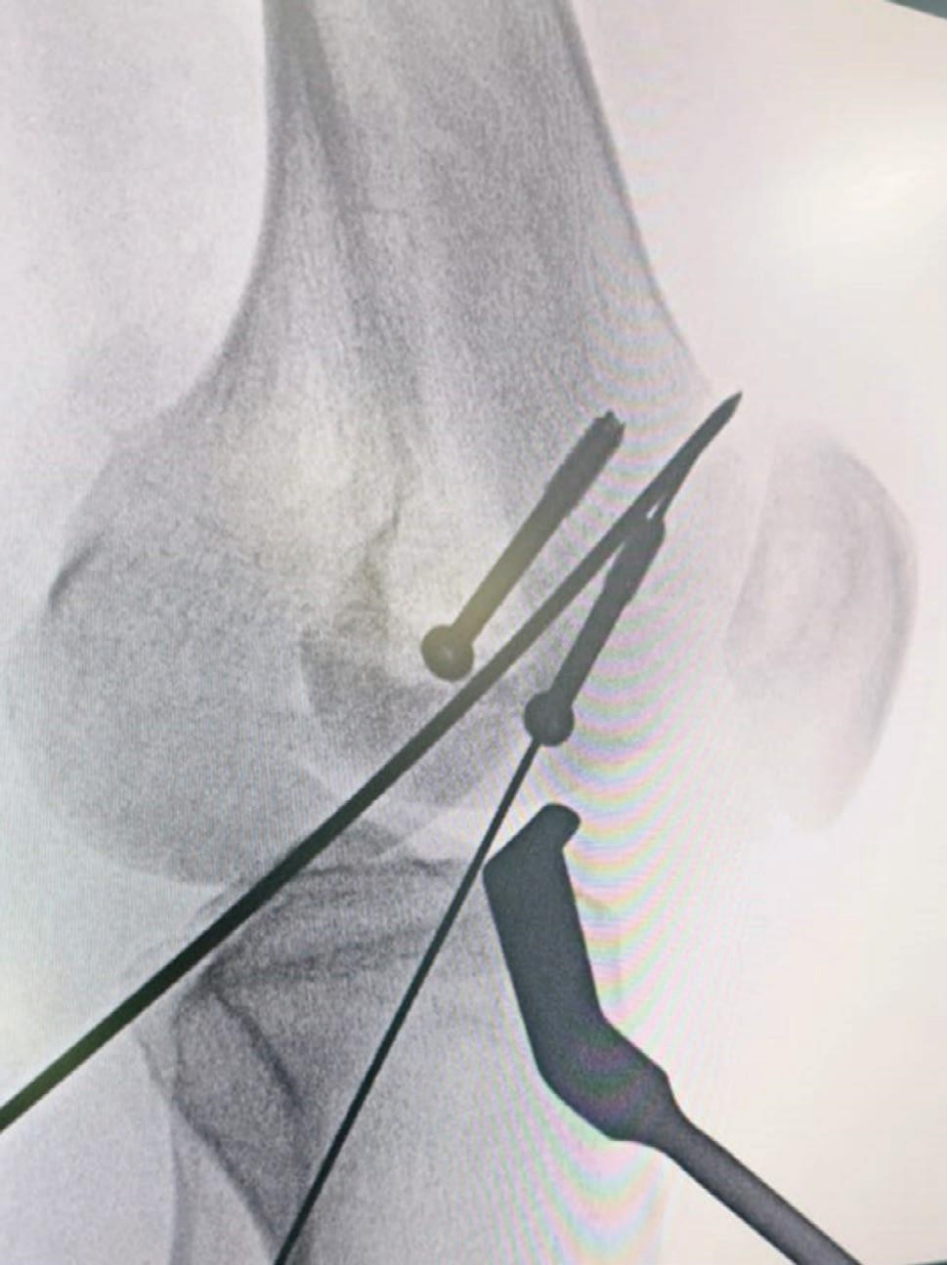
(b)

Postoperatively, the patient was kept nonweight bearing for 6 weeks, which applied to both legs due to the pelvic injury. During this period, the patient was encouraged to perform isometric quadriceps exercises, calf strengthening, and knee mobilization. Follow‐up appointments were scheduled at 2 weeks, 1, 3, and 6 months after surgery.

## 3. Results

There was a steady improvement in ROM and functional outcome on regular follow‐up. There was no extension lag, and knee flexion improved up to 90° at the 6‐week follow‐up. ROM further improved to 110° at 3 months and subsequently to 125° at the 6‐month follow‐up (Table 1). Neer knee score was used for functional evaluation [9]. This scoring involves both clinical and investigative parameters and is rated out of 100 (Table 2). A score above 85 is considered excellent, between 70 and 85 is considered satisfactory, between 55 and 69 is considered unsatisfactory, and a score below 55 is deemed a failure.

**Table 1:** Functional outcomes assessed using the Neer knee scoring system and knee flexion range of motion (ROM) at preoperative, 6‐week, 3‐, and 6‐month follow‐up intervals. *p* values represent paired comparison between preoperative and final follow‐up values.

	Preop	6 weeks	3 month	6 month	*p*‐Value
Neer score	30	74	84	90	0.0077
ROM—Flexion (degrees)	25	90	110	125	0.0144

**Table 2:** Neer knee scoring system used for functional evaluation [9]. In the present case, only total Neer scores were prospectively recorded at each follow‐up interval.

Parameters	Maximum scores	Criteria
1. Pain	**20**	No pain (20), intermittent pain (16), pain with fatigue (12), pain limiting function (8), and constant pain/night pain (4–0)
2. Walking capacity	**20**	Same as before (20), mild restriction (16), restricted/stairs sideways (12), and use of walking aids (4–0)
3. Joint movement	**20**	Normal/135° (20), up to 100° (16), up to 80° (12), up to 60° (8), up to 40° (4–0), and up to 20° (2)
4. Work capacity	**10**	Same as before (10), regular with handicap (8), altered work (6), light work (4), and no work (2–0)
5. Gross anatomy	**15**	Thickening only (15), 5° angulation/0.5 cm shortening (12), 10°/2 cm (9), 15°/3 cm (6), deformity (3), and nonunion/infection (0)
6. Roentgenogram	**15**	Near normal (15), 5°/0.5 cm displacement (12), 10°/1 cm (9), 15°/2 cm (6), union with deformity (3), and nonunion (0)

The patient′s functional outcome, as assessed by the Neer score, demonstrated significant improvement over time, increasing from 30 preoperatively to 90 at the 6‐month follow‐up, corresponding to an excellent result (*p* = 0.0077). Knee flexion ROM also showed progressive improvement, increasing from 25° preoperatively to 125° at the final follow‐up (*p* = 0.0144), indicating a substantial recovery in both function and mobility. In addition to functional assessment, radiological evaluation of fracture healing was performed at each follow‐up visit. Plain radiographs were obtained at 1, 3, and 6 months postoperatively. Follow‐up x‐rays demonstrated satisfactory fracture alignment and stable fixation, with no evidence of displacement, implant failure, or other complications (Figure 5).

Figure 5Postoperative radiographs of the right distal femur demonstrating fracture healing progress. (a) Anteroposterior (AP) view at 1 month, oriented with anterior structures at the top and medial structures to the left; (b) lateral view at 3 months, oriented with anterior structures to the right.

**Figure:**
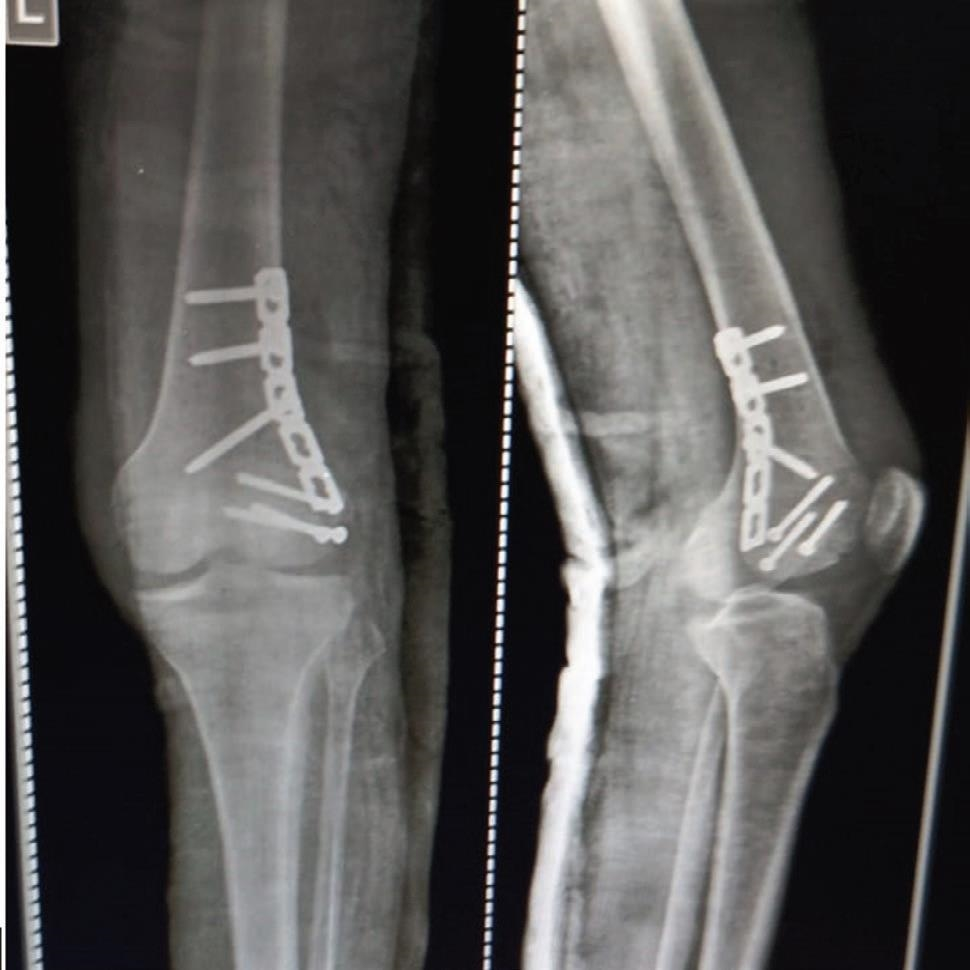
(a)

**Figure:**
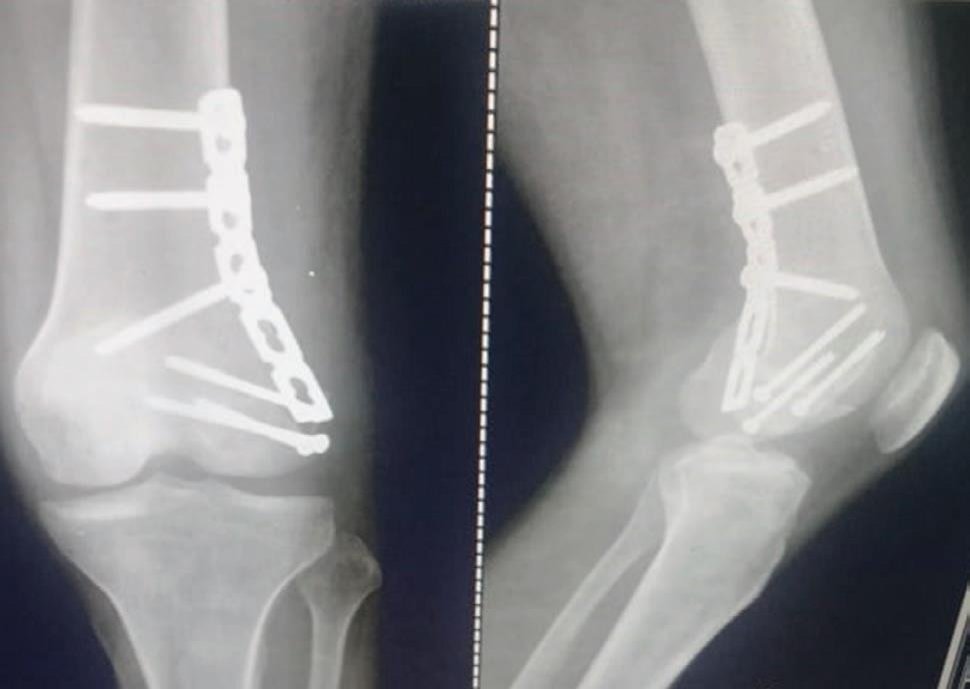
(b)

Statistical analysis was performed using a paired *t*‐test to compare preoperative and final follow‐up (6‐month) values for functional outcomes. A *p* value < 0.05 was considered statistically significant. Although the analysis is limited by the single‐patient nature of this case report, it was used to demonstrate the magnitude of functional improvement over time.

## 4. Discussion

Hoffa fractures are often overlooked unless the surgeon conducts a thorough examination. Despite clear clinical signs, radiographic detection can be challenging. These fractures may go unnoticed in standard anteroposterior views due to the intact anterior portion of the femoral condyle, and even lateral views may fail to reveal minimally displaced fractures. As a result, a CT scan is essential for accurately diagnosing and evaluating these fractures [8]. The purpose of classification systems is to describe the severity of fractures, assess the surgical expertise required, determine the preferred fixation method, and predict the final prognostic outcome. However, due to the rarity and complexity of Hoffa fractures, few classification systems have been proposed in the literature. The Muller AO/OTA classification recognizes these fractures as 33‐B3, but it offers limited insight into the diverse fracture patterns or their implications for prognosis and treatment [4]. Similarly, Letenneur′s classification, which is based on the orientation of the fracture line, does not adequately address these critical factors [6]. These fractures may exhibit patterns that do not neatly fit into any existing classification or may fall somewhere between the various categories. As a result, careful preoperative planning using CT imaging is crucial, though it can still present challenges for the surgeon.

Pires et al. [10] proposed a treatment algorithm based on a modified version of Letenneur′s classification, which incorporated a variant for cases of posterolateral shearing accompanied by an impacted or comminuted osteochondral fragment within the articular surface (Letenneur Type I variant). However, the authors acknowledged that neither did they utilize any functional scoring systems to assess the effectiveness of their protocol, nor did they provide data to support the reliability of the classification. In the CT classification proposed by Bagaria et al., the fracture in our study most closely aligns with their Type 1 classification, which refers to a unicondylar fracture with a fragment size greater than 2.5 cm. According to their protocol, such fractures should be treated with lag screws inserted in an anterior‐to‐posterior direction via a standard medial or lateral parapatellar approach. However, due to the unique nature of our fracture pattern, this approach was not feasible. Given the specific characteristics of the fracture, we opted for a posterolateral approach and inserted lag screws from posterior to anterior. This highlights the reality that, although classification systems can guide treatment, the surgeon must often adapt their approach based on the individual fracture pattern and its specific challenges.

In our case, preoperative CT planning revealed a unique fracture pattern, predominantly involving the distal articular surface of the posterior femoral condyle, without articular comminution. Initially, two lag screws were placed above the articular surface to protect the cartilage, but satisfactory reduction was not achieved. A third lag screw was inserted through the articular surface to obtain proper alignment, and a buttress plate was applied for additional stability. This approach ensured rigid fixation and effective reduction. Consistent with Gavaskar et al.′s [11] findings on Hoffa fractures, which emphasize anatomical reduction, rigid fixation, and early mobilization, our treatment method is aimed at achieving these goals while addressing the specific challenges of this atypical fracture.

## 5. Conclusion

In conclusion, Hoffa fractures present significant diagnostic and treatment challenges due to their atypical fracture patterns and the limitations of standard radiographic views. Although classification systems like Muller AO/OTA and Letenneur provide useful frameworks, they may not fully account for the diverse presentations of these fractures, requiring individualized surgical planning. Our case underscores the importance of careful preoperative CT assessment and highlights the need for flexibility in treatment strategies, as evidenced by our use of a posterolateral approach with posterior‐to‐anterior lag screws. By focusing on anatomical reduction, rigid fixation, and early mobilization, we aim to optimize recovery while addressing the unique complexities of this fracture type, in line with established treatment principles. Further research and refinement of classification systems may help improve outcomes for these challenging injuries.

## Funding

No funding was received for this manuscript.

## Ethics Statement

According to institutional policy, formal ethical approval is not required for single‐patient case reports. Written informed consent was obtained from the patient for publication of clinical details and images.

## Conflicts of Interest

The authors declare no conflicts of interest.

## Data Availability

The data that support the findings of this study are available on request from the corresponding author. The data are not publicly available due to privacy or ethical restrictions.
